# Decoding reappraisal and suppression from neural circuits: A combined supervised and unsupervised machine learning approach

**DOI:** 10.3758/s13415-023-01076-6

**Published:** 2023-03-28

**Authors:** Parisa Ahmadi Ghomroudi, Michele Scaltritti, Alessandro Grecucci

**Affiliations:** 1 Clinical and Affective Neuroscience Lab, Department of Psychology and Cognitive Sciences – DiPSCo, University of Trento, Rovereto, Italy; 2 Center for Medical Sciences – CISMed, University of Trento, Trento, Italy

**Keywords:** Reappraisal, Suppression, Machine learning, ICA, Boosted trees, Grey matter

## Abstract

Emotion regulation is a core construct of mental health and deficits in emotion regulation abilities lead to psychological disorders. Reappraisal and suppression are two widely studied emotion regulation strategies but, possibly due to methodological limitations in previous studies, a consistent picture of the neural correlates related to the individual differences in their habitual use remains elusive. To address these issues, the present study applied a combination of unsupervised and supervised machine learning algorithms to the structural MRI scans of 128 individuals. First, unsupervised machine learning was used to separate the brain into naturally grouping grey matter circuits. Then, supervised machine learning was applied to predict individual differences in the use of different strategies of emotion regulation. Two predictive models, including structural brain features and psychological ones, were tested. Results showed that a temporo-parahippocampal-orbitofrontal network successfully predicted the individual differences in the use of reappraisal. Differently, insular and fronto-temporo-cerebellar networks successfully predicted suppression. In both predictive models, anxiety, the opposite strategy, and specific emotional intelligence factors played a role in predicting the use of reappraisal and suppression. This work provides new insights regarding the decoding of individual differences from structural features and other psychologically relevant variables while extending previous observations on the neural bases of emotion regulation strategies.

## Introduction

Emotion regulation is now considered as a core construct for mental health. Deficits in emotion regulation may lead to psychological disorders (Ochsner and Gross, 2008; Kring and Sloan, 2009; Sheppes et al., 2015; Frederickson et al., 2018; Grecucci et al., 2020; Messina et al., 2021; Monachesi et al., under review). Evidence suggests that depression and anxiety (Gross and Muñoz, 1995; Campbell-Sills et al., 2006; Grecucci et al., 2020), bipolar disorder (Johnson 2005; Lapomarda et al., 2021a, b), and substance abuse disorder (Sher et al., 2007) may all be the result of severe emotion dysregulation. Difficulties in emotion regulation within social situations are a main feature of borderline personality disorder (Kring and Werner, 2004; Ochsner and Gross, 2008). In addition, aggression (Donahue et al., 2014), anger outbursts and sleep disorders (Gruber et al., 2009) are believed to stem from emotion dysregulation. Due to the pervasive occurrence of emotion regulation problems across psychological disorders, clinicians have started incorporating techniques to regulate emotions in their treatments (Linehan, 1993; Beauregard, 2007; Leahy et al., 2011; Messina et al., 2013; Dadomo et al., 2016, 2018; Frederickson et al., 2018; De Panfilis et al., 2019; Grecucci et al., 2020). Two widely studied emotion regulation strategies are reappraisal and suppression. Reappraisal is an antecedent-focused regulation strategy that modifies emotion before full activation of the emotional response. Reappraisal thus involves a voluntary attempt to reinterpret the meaning of a situation to alter its emotional impact (Gross, 1998). Differently, suppression is focused on the modification of emotions after their full activation and is defined as an attempt to inhibit an ongoing emotion-expressive behavior (Gross & Levenson, 1993). A few experimental studies examining the effect of reappraisal and suppression suggested that reappraisal decreases the behavioral expressions related to negative emotions and does not increase physiological responses compared with when no regulation is implemented (Gross, 2002, 2015; Goldin et al., 2019). In contrast, suppression results in a reduction of positive emotion experience, while leaving negative emotion experience unchanged and yielding a physiological impact (Gross & Levenson, 1993, 1997; Gross, 2002; Mauss et al., 2005; Brans et al., 2013).

Interestingly, individuals with more negative affect show a less habitual use of reappraisal and more frequent use of suppression whereas individuals with more positive affect tend to display the opposite pattern (more frequent use of reappraisal and a less habitual use of suppression) (Gross & John, 2003; John & Eng, 2014). Moreover, neuroticism seems to be associated with a reduced use of reappraisal and a more frequent reliance on suppression (John & Gross, 2004).

In the past 20 years, researchers have tried to understand the neural bases of specific emotion regulation strategies. One meta-analysis of 48 task-related fMRI studies focusing on reappraisal (Buhle et al., 2014) reported increased activation in bilateral dorsolateral and ventrolateral prefrontal cortex (dlPFC, vlPFC), dorsal anterior cingulate cortex (dACC), supplemental motor area (SMA), and inferior/superior parietal cortex during both down- and upregulation of emotion. A more recent meta-analysis of 42 fMRI studies on reappraisal and acceptance showed decreased activation of limbic areas, increased activity in dlPFC and left lPFC during reappraisal (Monachesi et al., under review). One study showed increased activity in prefrontal cortex (PFC) and decreased activity in the insula and in the amygdala during reappraisal in response to disgust-eliciting film clips (Goldin et al., 2008). In addition, a MRI study showed the habitual use of reappraisal is associated with higher activity in the fronto-cingulate cortex (Vanderhasselt et al., 2013). Importantly, another study revealed that reduced resting-state functional connectivity between right amygdala and medial PFC and posterior cingulate cortex (which are core components of the default mode network, DMN) predicted success in the use of reappraisal strategies (Uchida et al., 2015). Relatedly, two resting state functional connectivity studies showed that individual differences in reappraisal affect DMN’s functional connectivity (Martins and Mather, 2016; Morawetz et al., 2016). Finally, few studies suggest that the activity of DMN increases during reappraisal when viewing negative stimuli (Sripada et al., 2014; Xie et al., 2016). Previous studies have consistently linked DMN to semantic and autobiographical memory, thought generations, self-reflective processes, and cognitive elaboration of one’s affective state (Raichle et al., 2001; Crosson et al., 2002; Cato et al., 2004; Amodio and Frith, 2006; Olsson and Ochsner, 2008; Binder et al., 2009; Uchida et al., 2015). In line with these observations, we hypothesized that the role of DMN in reappraisal may be related to processes such as self-reflection and semantic elaboration of the meaning of the situation, to tame the affective response.

Concerning suppression, a study showed increased activity in the PFC, insula, and amygdala during suppression in response to disgust-eliciting film clips (Goldin et al., 2008). The habitual use of suppression has been associated with an increase in baseline perfusion of the medial PFC (Abler et al., 2008). Also, this tendency was associated with decreased activation of the orbital medial PFC while expecting to see unpleasant images (Abler et al., 2010). Moreover, a fMRI study showed the habitual use of suppression is associated with higher activity in amygdala (Vanderhasselt et al., 2013). One study on functional connectivity revealed a positive correlation between amygdala and dACC and a negative one between left centromedial amygdala and the SMA when using suppression (Picó-Pérez et al., 2018). Interestingly, one study by Sikka et al. (2022) further suggested that some brain regions involved in suppression (e.g., insula, frontoparietal, and inferior parietal cortex) may overlap with the salience network. In the same vein, Muhtadie et al. (2021) suggested that the salience network, especially the insular part, may be a major hub for emotional salience processing. Indeed, the activity of the insula has been found to be modulated during the down-regulation of unpleasant stimuli (Grecucci et al., 2013a, b). Such modulation has been interpreted as a modulation of the emotive arousal elicited by the stimuli. Indeed, information from different parts of the brain (such as the amygdala, the anterior cingulate cortex, and the hypothalamus) is integrated inside the insula to generate a model of the affective and proprioceptive state of the body. As such, we think the network encoding suppression may show a certain degree of overlap with the salience network. Suppression may act upon the map of the bodily/affective state generated at the level of the insula.

Beside functional task-related studies, researchers also have tried to understand how individual differences in emotion regulation strategies usage are mapped onto structural properties of the brain. Task-related activity provides real time information during cognitive and emotional operations (Poldrack and Gorgolewski, 2017). However, most fMRI studies consist of less than 50 participants, and often tasks vary across studies. Furthermore, for analyzing fMRI data, a large amount of information is required, such as the description of the task and the timings of the events. Therefore, combining task-based studies is more challenging compared to sMRI (Poldrack and Gorgolewski, 2017). Differently, structural brain properties and questionnaire measuring individual differences may represent a valuable and complementary alternative to cognitive tasks (Poldrack et al., 2017; Poldrack and Gorgolewski, 2017). In one sMRI study, a region of interest (ROI) analyses revealed a positive correlation between the dorsal anterior cingulate cortex volume and the use of reappraisal (Giuliani et al., 2011a). In another voxel-based morphometry (VBM) study, a positive correlation between reappraisal and right and left amygdala volume was found (Hermann, Bieber et al., 2013a). More recently, Pappaianni et al. (2020) conducted a study by using an unsupervised machine learning method based on Independent Component Analysis (ICA) known as source-based morphometry (SBM, Xu et al., 2009) to investigate the individual differences in structural brain features as a function of reappraisal usage. In this study, 37 participants were divided to low and high reappraisal groups as a function of their scores on the Emotion Regulation Questionnaire (ERQ; Gross & John, 2003). The results revealed higher concentration of gray matter in a network including the frontal, temporal, and parietal regions, among low reappraisers compared with high reappraisers. However, this study focused only on reappraisal, the sample size was quite small for machine learning analyses, and the comparisons were conducted between two subsamples instead of considering how individual differences are continuously mapped onto the brain.

Regarding suppression, a study showed a positive correlation between the right dorsomedial prefrontal cortex (dmPFC) volume and suppression usage (Kühn et al., 2011). In addition, a study using ROI and VBM analysis showed no relation between suppression use and volume of dACC (Giuliani et al., 2011a). Another study showed a positive correlation between anterior insula volume and use of suppression (Giuliani et al., 2011b). Finally, a VBM study conducted by Hermann et al. (2013a) found a positive correlation between suppression usage and dorsal anterior cingulate/paracingulate cortex and medial PFC grey matter volume.

The studies summarized above do not offer a clear-cut picture of the neural bases of reappraisal and suppression. This may be due to several methodological limitations: low number of participants, a priori defined ROI analyses, and massive univariate approaches. A perhaps more consistent picture can be found when examining the relations between the use of reappraisal vs suppression and some core psychological features. Different studies have suggested that some strategies may be more linked with specific emotional dysregulations (Dadomo et al., 2018; De Panfilis et al., 2019; Grecucci et al., 2020). For example, suppression has been associated with anxiety (Aldao and Nolen-Hoeksema, 2012), and some studies actually revealed that suppression may lead to the development of anxiety (Salters-Pedneault et al., 2006; Werner et al., 2011). By contrast, reappraisal is thought to be negatively associated with anxiety (Martin and Dahlen, 2005; Garland et al., 2011; Desrosiers et al., 2013; Peh et al., 2017). Beside anxiety, emotional intelligence (EI) may also play a role in the use of different regulation strategies. According to Mayer and Salovey (1997) EI is defined as “*the ability to perceive accurately, appraise, and express emotion; the ability to access and/or generate feelings when they facilitate thought; the ability to understand emotion and emotional knowledge; and the ability to regulate emotions to promote emotional and intellectual growth.*” Few studies have investigated the association between level of EI and use of reappraisal and suppression. One study has reported that a high score in EI was associated with a less frequent use of suppression (Andrei et al., 2016). In addition, other studies reported a positive association between EI level and the use of reappraisal together with a negative association between the use of suppression and EI (Schutte et al., 2009; Cabello et al., 2013; Nozaki, 2018). Consistently, a meta-analysis of 90 studies revealed that the individuals with higher levels of EI are more likely to use reappraisal whereas individuals with lower levels of EI are more prone to use suppression (Peña-Sarrionandia et al., 2015).

## Present study

Building on the considerations above, the purpose of the present study was to provide new evidence of how individual differences in the use of reappraisal and suppression can be predicted by structural properties of the brain. Also, the role of emotional intelligence, anxiety, and the use of other strategies will be jointly considered in the same predictive model. By considering these different classes of predictors together, one of our goals is to assess their relative contribution in predicting the use of different strategies of emotion regulation.

Given the fact that reappraisal and suppression are different in their supposed psychological mechanisms (Gross, 2002), we expect different brain regions to be involved. Building on previous studies on this topic (Pappaianni et al., 2020; Giuliani et al., 2011b; Kühn et al., 2011), we hypothesized that the individual differences in reappraisal usage involve a network consisting of cognitive, semantic, and top-down control regions, such as ventral medial PFC, parahippocampus, and temporal regions of the brain. One additional hypothesis is that the network predicting reappraisal may at least partially overlap with the DMN, as the latter has been previously associated with self-referential processes and self-generated thoughts (Andrews-Hanna, 2012; Andrews-Hanna et al., 2014), as well as with conceptual processing and perspective-taking. All these processes are thought to be involved in reappraisal. The prediction is that the greater the grey matter concentration inside this network, the greater the reappraisal usage. We also expect EI to have a positive association, and anxiety a negative association with the use of reappraisal.

By contrast, on the basis of previous studies (Giuliani et al., 2011a; Kühn et al., 2011; Hermann et al., 2013), we hypothesize that individual differences in the usage of suppression are related to a network including the insula, for its relationship with arousal and bodily awareness, and parietal-cerebellar regions more linked with response control and monitoring. We also predict this network to overlap with the salience network in which the insula is the main hub. The salience network may be related to the generation and modulation of the affective bodily map generated during emotional experiences. Here, the prediction is that the greater the grey matter concentration in this network, the greater the suppression usage. We also expect EI to have a negative association, and anxiety to have a positive association, in predicting the frequency of usage of suppression. Moreover, together with the psychological features, in the final model we also will include the ERQ score of the other strategy as a predictor (suppression for reappraisal, and reappraisal for suppression). Previous studies (Benson et al., 2019) suggested that the usage of one strategy may be predictive of usage of the other. Building on this, we expect individuals who frequently use one strategy to frequently also use the other.

In the present study, we incorporated these psychological features (together with the structural brain features) in the same predictive models to assess their relative contribution in a cohesive predictive model jointly capturing structural brain measures and psychological features. From a methodological point of view, a combination of unsupervised and supervised machine-learning algorithms was used with a twofold purpose. First, we aimed to decompose the brain into naturally grouping independent grey matter circuits using ICA, an unsupervised machine learning approach. Second, we aimed to predict individual differences in the use of reappraisal and suppression by using the independent circuits of the first analysis by using a regression-based supervised machine learning approach (boosted decision trees) and including EI and anxiety scores as predictors. We believe that this approach may display several advantages or, at least, an important and complementary source of information concerning the issues at stake. ICA is a multivariate method that considers the statistical dependency among voxels (Xu et al., 2009). Accordingly, the brain is separated into independent brain circuits based on regions with covarying grey matter concentration. In other words, the brain is decomposed into naturally grouping networks with lower and consistent dimensionality (Grecucci et al., under review). Such approach also is more coherent with a network perspective in neuroscience (Hamann, 2012).

Additionally, previous studies used correlations or regressions to find association between networks parameters and emotion regulation variables. One limit of such frequentist approach is that results are strictly dependent on the sample and cannot be generalized to new cases. In the current paper, we used a supervised machine learning algorithm to see which structural features of the brain networks better predicts the use of reappraisal or suppression within new cases. In other words, the statistical model derived from the training sample is then tested on a different subset of data to assess generalization to unobserved cases (hold-out method). As such, this model directly tackles the predictive ability of the variables considered.

## Method

### Participants

Brain scans and questionnaires scores of 135 participants were included in the present study. The data were selected from “Leipzig study for mind-body-emotion interactions” (OpenNeuro database, accession number ds000221, LEMON) and were collected at the Max Planck Institute for Human Cognitive and Brain Sciences (MPI CBS) in Leipzig (Babayan et al., 2019). The participants were prescreened by telephone interviews. The exclusion criteria for data collection were as follows: no cardiovascular disease, history of psychiatric diseases, history of neurological disorders, history of malignant diseases, intake of the following medications: centrally active medication, beta- and alpha-blocker, cortisol, any chemotherapeutic, or psychopharmacological medication. We extracted a subset of participants with negative drug test and no alcohol use. Seven participants of the original sample of 135 were excluded due to corrupted data. Therefore, the final number of participants was 128 (36 females) with mean age 29.72 ± 12.43 and average 12.73 ± 0.87 years of education. Participants provided written, informed consent, and they agreed to their data being shared anonymously. Participants received compensation for participating in the study after the completion of all assessments.

### Image acquisition

Structural images were acquired using a 3 Tesla scanner (MAGNETOM Verio, Siemens Healthcare GmbH, Erlangen, Germany) equipped with a 32-channel head coil.

### Behavioral data

To address our experimental questions, beside sMRI data, scores from three questionnaire were considered. The German version of the ERQ (Abler and Kessler, 2009) was selected to measure the frequency of usage of reappraisal and suppression. This questionnaire consists of ten questions. Six of the questions measure the tendency to use reappraisal and four questions measure suppression. Each response is on 7-point Likert-type scale ranging from 1 (strongly disagree) to 7 (strongly agree). The German version (Laux et al., 1981) of State-Trait Anxiety Inventory (STAI-G-X2, Spielberger et al., 1970) was used to assess the trait anxiety levels of participants. It consists of 20 questions on a 4-point Likert scale ranging from 1 (almost never) to 4 (nearly always). Finally, the German adaptation (Freudenthaler et al., 2008) of Trait Emotional Intelligence Questionnaire-Short Form (TEIQue-SF, Petrides and Furnham, 2006) was used to assess the EI of the participants, and its subscales Well-Being, Self-Control, Emotionality, and Sociability. This questionnaire consists of 30 items, including two items from each of the 15 facets of the TEIQue.

### Data analysis

#### Pre-processing

First, the quality of structural MRI data was assessed to exclude any possible artifacts. Data were then pre-processed using Computational Anatomy Toolbox (CAT12, http://www. neuro.uni-jena.de/cat/), a toolbox for statistical Parametric Mapping (SPM12) in MATLAB environment (The Mathworks, Natick, MA). The structural images were manually reoriented to the anterior commissure as the origin. Then, the images were segmented into grey matter (GM), white matter (WM), and cerebrospinal fluid (CSF) using CAT12. Next, the GM image registration was conducted with Diffeomorphic Anatomical Registration using Exponential Lie algebra (DARTEL) tools for SPM12 instead of traditional whole brain registration (Yassa and Stark, 2009; Grecucci et al., 2016; Pappaianni et al., 2018) Finally, the DARTEL images were normalized to MNI-152 standard space and each image were smoothed with a 12-mm, full-width at half-maximum (FWHM) Gaussian kernel [12, 12, 12].

#### Unsupervised machine learning to decompose the networks

ICA was applied to the structural MR images of participants to identify independent circuits across the whole brain (Xu et al., 2009; Pappaianni et al., 2018; 2020; Sorella et al., 2019; Saviola et al., 2020; Lapomarda et al., 2021a). ICA, part of Blind Sources Separation methods (Karhunen and Malaroiu, 1999), is an unsupervised machine learning procedure, which can be used to decompose the brain into naturally grouping networks based on covariations in the grey matter concentration. The resulting circuits represent specialized and partially segregated networks. In this study, the GroupICA toolbox (GIFT, http://mialab.mrn.org/software/gift/) was used inside MATLAB environment (The Mathworks, Natick, MA). Following default parameters, 20 independent components were extracted. Infomax algorithm was used to minimize the mutual information of the network outputs (Bell and Sejnowski, 1995; Lee et al., 1999). Then, we selected ICASSO, a GIFT toolbox to investigate the reliability of the ICA algorithm. RandInit and Bootstrap were selected in ICASSO to provide stability of the components (Kubera et al., 2014). As suggested by the authors, ICA was set to run 100 times, and the minimum and maximum cluster sizes were set at 80 and 100 respectively. ICA returned a matrix with the number of participants (row) and a vector of loading coefficients (columns), the columns indicate how each network is expressed in every participant. These loading coefficients were then entered into supervised machine learning to see which circuits correctly predict the use of reappraisal and suppression.

#### Supervised machine learning to build a predictive model

The loading coefficients found by ICA were entered into supervised machine learning to predict the use of reappraisal and suppression. The MATLAB Statistical and Machine Learning toolbox was used to conduct such analysis. The purpose of this analysis was twofold: to build a model that correctly predicts reappraisal and suppression usage, and to allow generalization of our results to predict new cases. The feature selected to build a predictive model for reappraisal usage were: STAI, TEIQue-SF subscales, ERQ suppression scores, and the ICs loading coefficients. To build a predictive model for suppression usage, the same predictors were selected, except for ERQ suppression scores, which were used instead of the reappraisal ones. Specifically, we used boosted regression tree model, which involves two techniques, namely decision tree algorithms and boosting methods. Decision trees are fit to improve the accuracy of the model and the boosted decision tree trains the model. Boosting algorithm is an adaptive method for combining many trees to improve the predictive model. Specifically, boosted regression trees are additive regression models in which simple trees are fitted in a forward, stepwise fashion. The error in each tree is calculated by a loss function and it is corrected in the next tree. Boosted regression trees have a number of advantages over tree-based methods. First, this model handles different types of predictors and accommodates missing data. In addition, prior data transformation or elimination of outliers are not required. Finally, fitting multiple trees solves the relatively poor predictive performance (Elith et al., 2008).

#### Further analyses

**Fig. 1:**
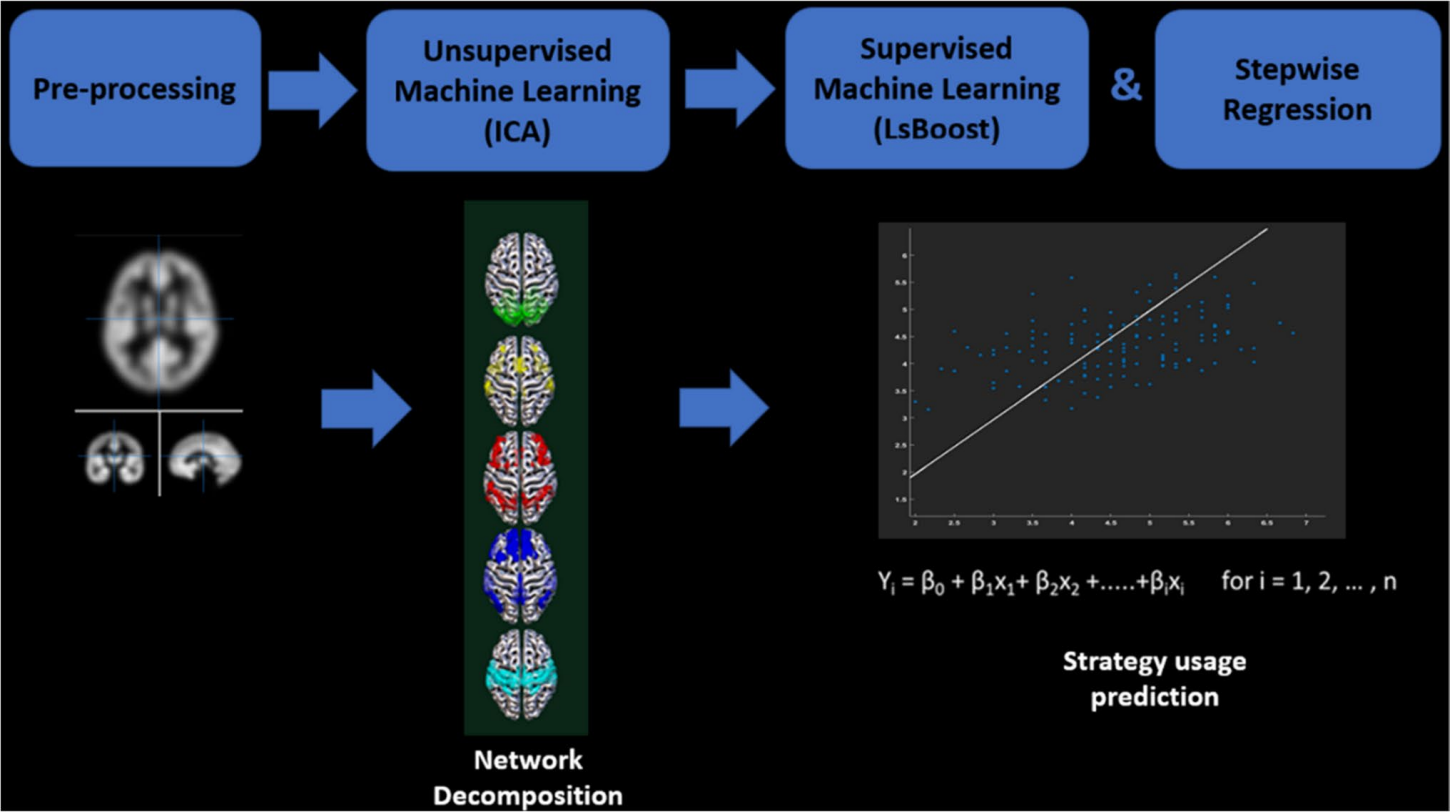
Schematic diagram of the methodology. First the T1 weighted images were preprocessed. Then 20 independent components were extracted using an unsupervised machine learning approach (ICA). Finally, the prediction model for reappraisal and suppression usage was obtained via a supervised machine learning method (boosted decision trees) and stepwise regression.

To check the consistencies of our results and to further explore the direction of the effects (not visible with the boost trees method), we also entered the predictors (ERQ-, STAI-, tEI-scores, loading coefficients of the ICs) in two separate stepwise regressions, one for each strategy to test whether they converge with the previous machine learning results. The regression module of JASP Team (2021). JASP (Version 0.16.0) was used to this aim (Fig. 1).

## Results

### Unsupervised machine learning

**Fig. 2:**
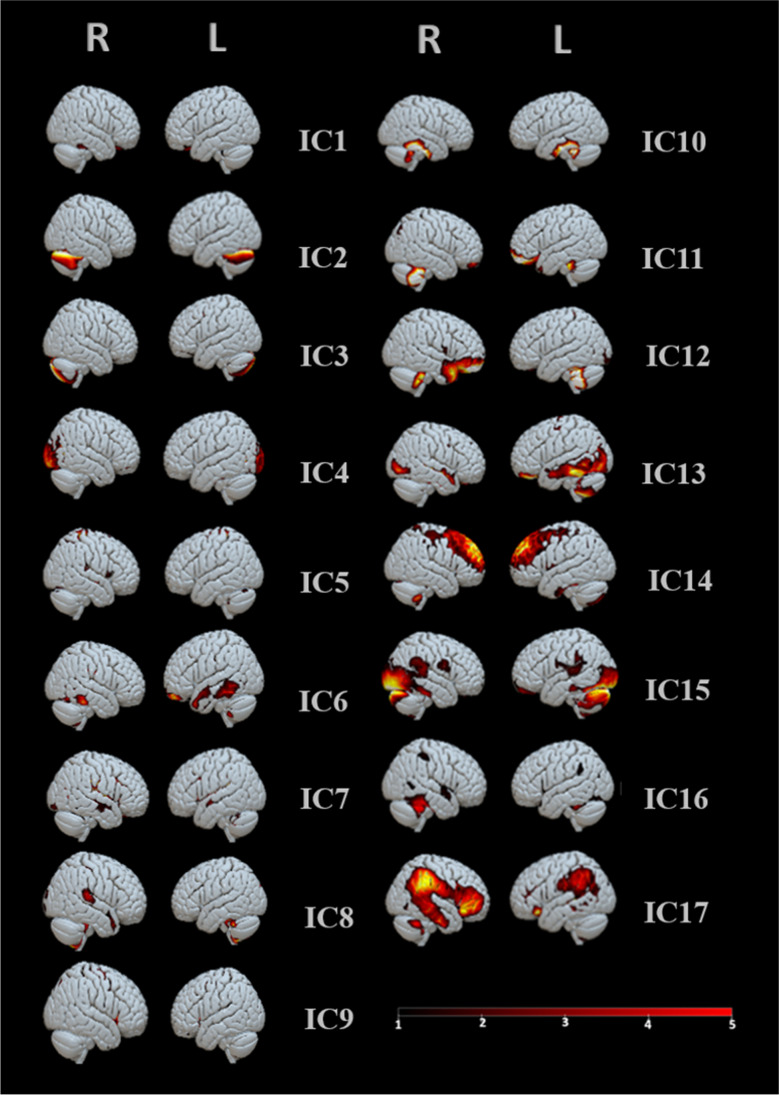
Independent components from 1 to 17. ICA was able to decompose the brain into 20 covarying grey matter networks. Three ICs were excluded for their reliability (Iq<0.9)

ICA was applied to structural data (Xu et al., 2009; Pappaianni et al., 2018, 2020; Sorella et al., 2019; Saviola et al., 2020; Lapomarda et al., 2021a) and returned a matrix containing 128 rows (number of participants) and 20 columns (number of independent components). The number of components was suggested as default number by GIFT. Only ICs with quality index (Iq) > 0.9 were included (from IC1 to IC17) for further analysis. The other components (IC18,19,20) were excluded (Fig. 2).

### Supervised machine learning

**Fig. 3:**
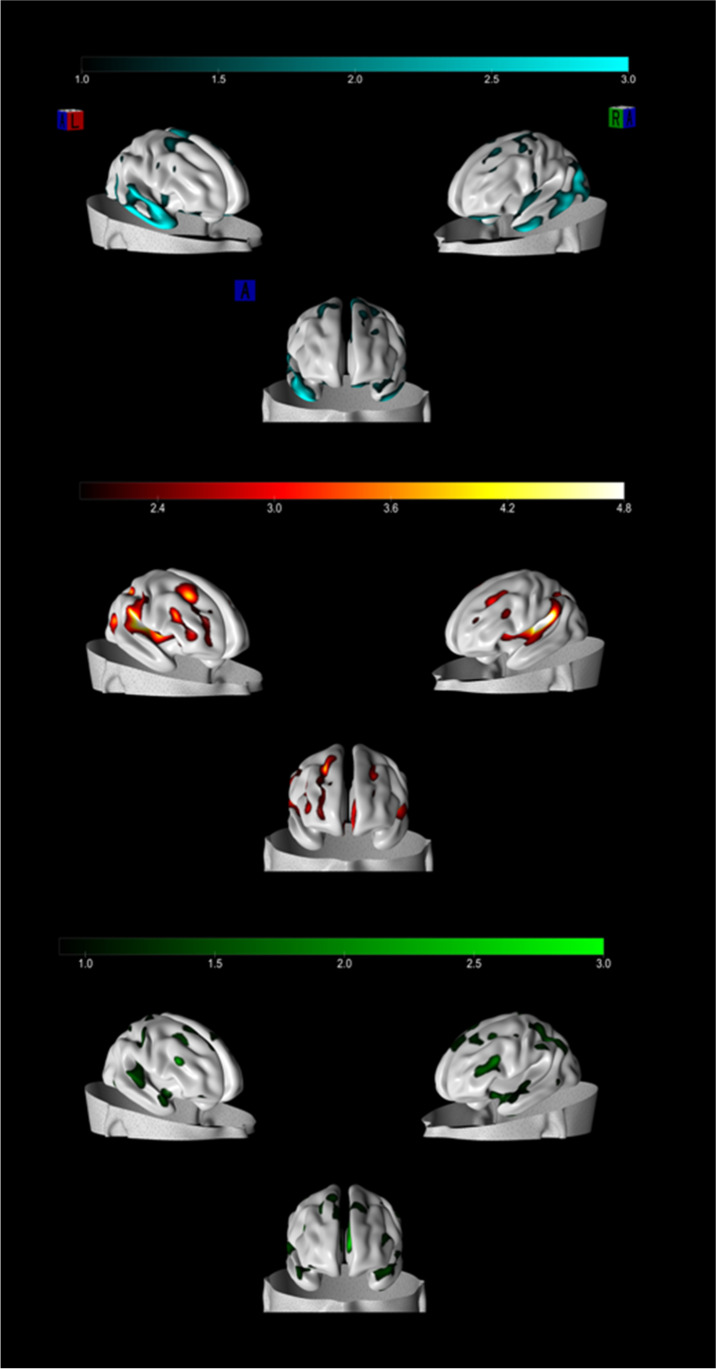
Top: brain plot reconstruction of C13 predicting reappraisal. Central part: brain plot reconstruction of IC7 predicting suppression. Bottom: brain plot reconstruction of IC8 predicting suppression

The statistical and machine learning toolbox of MATLAB (The Mathworks, Natick, MA) built a predictive model for reappraisal and suppression usage. In this analysis, the boosted tree algorithm was chosen. The result revealed that suppression score, IC13 (temporo-parahippocampal-orbitofrontal network), STA1 score, and tEI (all subscales) were relevant features in the boosted tree model for reappraisal usage. Moreover, Reappraisal Score, IC7 (insular network), STA1 score, tEI (all subscales) were relevant features in the boosted tree model predicting suppression usage (Fig. 3; Tables 1, 2, and 4).

**Table 1:** Winning models, IC13 for reappraisal and IC7 for suppression usage

Target	Features	Model	R^2^	RMSE	MSE	MAE	MLS	NoL	LR
RS (ERQ)	SS,STA1S, IC13, Tei SS	BT	0.32	0.8489	0.7206	0.6786	8	30	0.1
SS (ERQ)	RS,IC7, STA1S, Tei SS	BT	0.16	1.0315	0.9522	0.764	8	30	0.1
SS (ERQ)	RS, IC8, STA1 S, Tei SS	BT	0.12	1.0068	1.0136	0.7931	8	30	0.1

We additionally report the IC8 as the second winning model for suppression as confirmed by stepwise regression.

RS = reappraisal score; SS = suppression score; R^2^ = coefficient of determination; RMSE = standard deviation of the residuals; MSE = mean squared error; MAE = mean absolute error; MLS = minimum leaf size; NoL = number of learners; LR = learning rate; BT = boosted tree; Tei SS = Tei subscales; STAI S = STAI score.

**Table 2:** IC features for reappraisal and suppression, R-squared and RMSE values ordered from best to worse

Target	Feature	R^2^	RMSE	Target	Feature	R^2^	RMSE
Reappraisal (ERQ) Score	IC13	0.32	0.8489	Suppression (ERQ) Score	IC7	0.16	0.97582
	IC8	0.09	0.97268		IC8	0.12	1.0068
	IC1	0.08	0.9654		IC4	0.08	1.0199
	IC16	0.08	0.97462		IC3	0.07	1.0255
	IC4	0.06	0.97561		IC5	0.06	1.0315
	IC5	0.06	0.98135		IC13	0.06	1.046
	IC10	0.06	0.97761		IC17	0.05	1.0415
	IC7	0.05	0.99257		IC15	0.04	1.0487
	IC12	0.05	0.98817		IC9	0.02	1.066
	IC6	0.04	0.98159		IC14	0.02	1.0566
	IC11	0.04	1.0079		IC6	0.01	1.0608
	IC2	0.03	0.99897		IC2	0.01	1.0641
	IC15	0	1.0109		IC12	-0.01	1.0848
	IC3	-0.04	1.0256		IC10	-0.01	1.0679
	IC17	-0.04	1.0257		IC16	-0.07	1.1128
	IC14	-0.08	1.0621		IC11	-0.08	1.1224
	IC9	-0.09	1.0504		IC1	-0.15	1.1426

R^2^ = coefficient of determination; RMSE = standard deviation of the residuals.

### Further analysis

To ensure the goodness of the boosted regression trees and to find a possible convergence across different methods, we also used regression to predict reappraisal and suppression scores. This method allows to estimate the weight of each factor in predicting the variables of interest. Multiple linear regression using stepwise data entry showed that STAI, ERQ-suppression, tEI (wellbeing subscale) and IC13 (temporo-parahippocampal-orbitofrontal network) significantly predicted reappraisal usage F (4,123) = 11.404, *p* < 0.001 (Bonferroni corrected threshold), following the equation: reappraisal usage = 3.758 − (0.038 * STAI) + (0.277 * IC13) + (0.250 * ERQ (suppression score) + (0.230 * Tei_wellbeing). Multiple linear regression using stepwise data entry showed that STAI, ERQ-reappraisal scores, tEI (emotionality subscale), and IC8 (frontopariatal and cerebellar network) significantly predicted suppression scores F (4,123) = 6.926, *p* < 0.001 (Bonferroni corrected threshold), following the equation: suppression usage = 3.405 + (0.024 * STAI) − (0.235 * IC8) + (0.264 * ERQ reappraisal score) − (0.304 * tEI_emotionality). Moreover, the result depicted that reappraisal and suppression strategies covary (Fig. 3; Tables 3 and 4).

**Table 3:** Result from stepwise regression analysis with winning models IC13 for reappraisal and IC8 for suppression usage

Variable	β	SE	t	*p*	95% CI
STAI_Trait_Anxiety	−0.038	0.011	−3.525	<0.001	[−0.059, −0.017]
ICA 13	0.277	0.078	3.54	<0.001	[0.122, 0.432]
ERQ_suppression	0.25	0.075	3.347	0.001	[0.122, 0.432]
TeiQueSF_well_being	0.23	0.104	2.201	0.03	[0.122, 0.432]
TeiQueSF_emotionality	−0.364	0.11	−3.318	0.001	[−0.581, −0147]
ICA 8	−0.235	0.087	−2.692	0.008	[−0.408, −0.062]
ERQ_reappraisal	0.264	0.093	2.852	0.005	[0.081, 0.448]
STAI_Trait_Anxiety	0.024	0.012	2.103	0.037	[0.001, 0.047]

SE = standard error.

**Table 4:** Independent Component 13, 7, 8

Network	Area	Brodmann area	Volume (cc)	Random effects: max value (x, y, z)
Reappraisal (IC13)	Inferior temporal gyrus	20, 37	0.5/2.1	5.1 (−40, −12, −27)/7.1 (53, −29, −17)
	Sub-gyral	20	0.4/2.3	4.7 (−40, −16, −21)/6.8 (50, −31, −16)
	Fusiform gyrus	20, 37	0.4/2.3	5.2 (−40, −14, −25)/6.6 (40, −11, −26)
(IC13)	Middle temporal gyrus	20, 21	0.8/1.3	4.2 (-58, -51, 0)/6.5 (39, −6, −31)
	Uncus	20, 34	0.2/0.4	4.2 (−37, −14, −27)/5.9 (36, −7, −29)
	Cerebellar tonsil	*	0.0/1.4	−999.0 (0, 0, 0)/5.1 (25, −56, −38)
	Parahippocampal gyrus	20, 28, 34, 36	0.0/0.6	−999.0 (0, 0, 0)/4.9 (13, −5, −17)
	Superior temporal gyrus	22	0.0/0.6	−999.0 (0, 0, 0)/4.5 (52, −29, 6)
	Lentiform nucleus	*	0.0/0.3	−999.0 (0, 0, 0)/4.3 (28, −9, 2)
	Extra-nuclear	*	0.0/0.2	−999.0 (0, 0, 0)/3.9 (31, −6, 0)
	Rectal gyrus	11	0.2/0.0	3.6 (−9, 32, −26)/−999.0 (0, 0, 0)
	Thalamus	*	0.1/0.0	3.5 (−18, −19, 8)/−999.0 (0, 0, 0)
Suppression (IC7)	Insula	13, 40, 41	6.0/6.0	8.1 (−40, −28, 18)/7.1 (45, −25, 19)
	Superior temporal gyrus	13, 22, 41, 42	1.4/0.7	7.3 (−43, −28, 15)/6.0 (48, −27, 17)
	Sub-gyral	*	0.4/0.4	6.6 (−40, −31, 22)/5.5 (42, −28, 24)
(IC7)	Transverse temporal gyrus	41	1.2/0.4	6.6 (−40, −29, 12)/5.2 (45, −20, 12)
	Inferior parietal lobule	40	2.4/1.6	6.4 (−43, −28, 22)/6.4 (46, −28, 22)
	Postcentral gyrus	2, 40, 43	0.3/1.7	5.0 (−49, −25, 18)/6.2 (52, −24, 18)
	Extra-nuclear	*	0.1/0.4	4.1 (−34, −28, 24)/5.3 (40, −21, 22)
	Claustrum	*	0.0/0.1	−999.0 (0, 0, 0)/4.6 (37, −5, 7)
	Middle frontal gyrus	8, 46	0.0/0.4	−999.0 (0, 0, 0)/4.2 (24, 22, 42)
	Supramarginal gyrus	40	0.2/0.0	4.0 (−49, −39, 30)/−999.0 (0, 0, 0)
	Precentral gyrus	6, 13, 44	0.1/0.4	3.6 (−45, −8, 6)/3.9 (49, −7, 7)
	Middle temporal gyrus	19, 39	0.0/0.2	−999.0 (0, 0, 0)/3.9 (45, −63, 18)
	Superior parietal lobule	7	0.0/0.1	−999.0 (0, 0, 0)/3.7 (28, −50, 43)
	Medial frontal gyrus	25	0.1/0.2	3.6 (−1, 27, −13)/3.6 (4, 25, −18)
	Superior frontal gyrus	8	0.0/0.1	−999.0 (0, 0, 0)/3.6 (21, 25, 43)
Suppression (IC8)	Cerebellar tonsil	*	2.8/2.4	11.5 (−3, −56, −38)/11.2 (3, −56, -38)
	Inferior semilunar lobule	*	2.4/1.7	11.3 (−3, −59, −40)/11.0 (3, −59, −40)
	Fourth ventricle	*	0.1/0.3	4.8 (−3, −52, −25)/9.9 (0, −53, −33)
	Nodule	*	1.2/1.2	8.4 (−3, −52, −30)/8.5 (0, −55, −30)
	Uvula of vermis	*	0.3/0.2	7.4 (−3, −61, −32)/7.0 (3, −61, −32)
	Uvula	*	0.8/0.4	5.3 (−6, −67, −34)/6.4 (0, −61, −30)
	Culmen	*	3.2/0.4	5.0 (−30, −52, −23)/3.8 (28, −52, −21)
	Declive	*	0.4/0.0	4.3 (−27, −59, −21)/−999.0 (0, 0, 0)
	Pyramis of Vermis	*	0.1/0.0	3.5 (−3, −70, −29)/−999.0 (0, 0, 0)

Talairach labels of regions of interest, Brodmann area, volume (expressed in cc) and max values coordinates are shown

## Discussion

The purpose of the current study was to provide new evidence of how individual differences in the use of two different strategies of emotion regulation, reappraisal vs. suppression, can be predicted by stable structural features of the brain and by relevant psychological features, such as anxiety and emotional intelligence. We first applied an unsupervised machine learning algorithm to sMRI scans of 128 healthy individuals to decompose the brain into naturally grouping independent grey matter circuits (ICs). The unsupervised machine learning algorithm returned 17 distinct brain networks. Among these, IC13 captured a higher grey matter concentration within a temporo-parahippocampal-orbitofrontal network and was predictive of the use of reappraisal. Differently, IC7 reflected a higher concentration of gray matter within an insular network and was predictive of suppression. Notably, the subsequent stepwise regression analysis confirmed the role of IC13 (together with the other psychological variables considered) for reappraisal, whereas IC8, capturing the network of higher gray matter concentration within fronto-temporo-cerebellar regions (together with the other psychological variables considered), appeared to predict the use of suppression. Although the IC8 was not the component highlighted in the winning model (i.e., the model with higher R and lower RMSE; Table 2) of the boosted trees, it was the second component in order of importance just after the IC7. So, we consider both good candidates for the neural bases of suppression. In the following sections, we discuss our results in detail.

### Temporo-Parahippocampal-orbitofrontal network for reappraisal

The supervised machine learning approach and the stepwise regression both provide converging evidence that IC13 predicts individual differences in the use of reappraisal. IC13 is mainly composed of temporal, parahippocampal, and orbitofrontal regions. Concerning temporal regions, evidence from various neuroimaging studies converges on the importance of these areas in semantic and linguistic processes, particularly with reference to the lateral and ventral temporal cortex (middle temporal, inferior temporal, fusiform, and parahippocampal gyri) in reappraisal implementation (Ochsner & Gross, 2005, 2007; Ochsner et al., 2004, 2012; Buhle et al., 2014; Forseth et al., 2018).

Considering the role of the frontal regions, a greater success with the use of reappraisal has been shown to be positively correlated with resting state functional connectivity between the right amygdala and the left ventrolateral PFC (Morawetz et al., 2016) and negatively correlated with the functional connectivity between the right amygdala and the medial PFC (Uchida et al., 2015). Concerning the involvement of this frontotemporal network, it nicely dovetails with a well-established psychological model of emotion regulation (Ochsner and Gross, 2008), suggesting that prefrontal control regions may intervene to regulate and adjust semantic and perceptual representations of the stimuli in lateral-temporal regions during reappraisal (Ochsner & Gross, 2005, 2007; Ochsner et al., 2012; Messina et al., 2015, 2016). Another frontal region that is considered pivotal for successful reappraisal is the orbitofrontal cortex (Wager et al., 2008). In fact, a few studies suggest it plays a role in cognitive control functions that are critical for reappraisal, such as inhibition (Ochsner et al., 2004; Banks et al., 2007; Kanske et al., 2011). It is further interesting to note that recent evidence highlights the relevance of the connectivity between the OFC and the amygdala—a critical region for emotion processing—in the context of reappraisal. For example, Gao et al. (2021) have showed that the functional coupling between orbitofrontal cortex and amygdala is associated with use of reappraisal. Also, Kanske and colleagues (2021) showed decrease activity in the amygdala and increased activity in the orbitofrontal cortex during reappraisal. Possibly, these links between OFC and amygdala are an important neural underpinning of emotional regulations strategies and reappraisal in particular.

Concerning the parahippocampal gyrus, evidence suggests that this area plays a key role in memory processes, including coding and retrieval, as well as in emotional processes (Hamann, 2001; Gosselin et al., 2006; Van den Stock et al., 2012; Frank et al., 2014). In particular, according to Deak et al. (2017), increase activity in the parahippocampal area during reappraisal may be due to the semantic processes recruited when shaping a different interpretation of the same context. Consistently, a few studies have shown a link between parahippocampus and amygdala, in terms of functional connectivity. This link possibly mediates the connection between semantic/contextual processing and emotion (Aminoff et al., 2013; LaBar & Cabeza, 2006).

As expected, this temporo-parahippocampal-orbitofrontal network (IC13) partially overlaps with the DMN. The DMN includes brain regions that are active when individuals are not engaged in a specific task or during self-referential processes and self-generated thoughts (Andrews-Hanna, 2012; Andrews-Hanna et al., 2014). Core regions of the DMN indeed consist of posterior cingulate cortex, parts of the precuneus, medial PFC, bilateral inferior parietal lobule, and parts of the posterior temporal areas. In addition, hippocampus, medial temporal lobe, lateral temporal cortex and temporal pole also are often believed to be part of the DMN (Buckner et al., 2008; Broyd et al., 2009; Andrews-Hanna et al., 2010; Power et al., 2011). Interestingly, the dorsal medial PFC, the angular gyrus, the middle temporal gyrus, and the anterior temporal region that are part of DMN are involved in semantic processing (Binder et al., 2009; Wirth et al., 2011). The DMN may be involved in conceptual processing, perspective-taking, and reasoning; all these processes are crucial in reappraisal (Buhle et al., 2014). In conclusion, the greater the grey matter concentration inside this network, the greater these underlying abilities and, thus, the larger the reappraisal usage.

### Insular network and Fronto-parietal-cerebellar network predict suppression

The supervised machine learning approach returned the IC7 to be the best neural predictor of suppression usage. This network involves the insula and other key regions. The insula receives and combines inputs from various limbic and cortical regions, such as the amygdala the anterior cingulate cortex and the orbitofrontal cortex. The integration of these regions produces a coherent model of self that consist of bodily states (Craig, 2002; 2009; 2010), which are essential elements when asked to suppress our emotions. Notably, a previous experiment on the regulation of emotions elicited during interpersonal situations reported significant modulation of the insula during emotion regulation (Grecucci et al., 2013a; b). These authors hypothesized that the modulation of activity at the level of the insula may represent the regulation of the emotion-driven physiological arousal.

For what entails the frontal regions included in IC7, Hayes et al. (2010) highlighted the association between the activation of the middle frontal gyrus and the ability to suppress facial expressions. Hence, the increased grey matter concentration in this area for frequent users of suppression strategies might stem from an enhanced awareness of facial expression. In addition, this network included superior temporal regions and the middle temporal gyrus. These areas may be involved in mentalizing, especially with reference to the awareness of others’ intentions through the decoding of facial expressions or head and body motion (Frith and Frith, 2003; Dörfel et al., 2014). Remarkably, this network greatly overlap with the so-called salience network, which indeed involves the insula, but also portions of the dorsal anterior cingulate cortex, the amygdala, the ventral striatum, and the substantia nigra/ventral tegmental area (Seeley et al., 2007; Menon and Uddin, 2010). Suppression relies on the integration of interoceptive awareness, proprioceptive awareness, social awareness, and personal salience (Muhtadie et al., 2021). The salience network is active during salient emotional stimuli, social behavior, and self-awareness (Craig, 2009; Menon and Uddin, 2010; Gogolla et al., 2014). For these reasons, the salience network may play a role in the use of suppression. A representation of the state of the body as encoded in the insula (Muhtadie et al., 2021) may be necessary for suppression to modulate the bodily affective state (Grecucci et al., 2013a, b). Again, the greater the grey-matter concentration inside this network, the greater these abilities, thus the enhanced use of suppression.

Stepwise regression revealed IC8 to play a major role in predicting the use of suppression, although with a negative relation. This circuit also was highlighted by the supervised machine-learning analysis (just after the IC7 for importance). IC8 consists of a large frontopariatal and cerebellar network. Frontal regions of the brain are considered to be very important for cognitive strategies of emotion regulation (Buhle et al., 2014; Kohn et al., 2014) but also in coordinating motor areas and in general cognitive monitoring of other functions (Aron et al., 2014; DePue et al., 2016). Moreover, frontopariatel regions have been shown to support attentional control functions (Cole et al., 2013, 2014; Dodds et al., 2011; Power et al., 2011; Scolari et al., 2015). The negative relation between such cognitive control/attentional regions (IC8) and suppression may be interpreted as indicating that suppression may rely less on top-down control mechanisms, which are instead pivotal when deploying cognitive control and attentional strategies. Both attention and cognitive control would thus not be involved in suppression. Building on this finding, one can conclude that the lesser the grey matter concentration inside this network, the lesser cognitive control abilities displayed, the larger the usage of suppression.

### Emotional intelligence and anxiety in predicting reappraisal and suppression

Our analyses (both boosted trees and stepwise regression) confirmed the additional role of anxiety and EI in predicting the use of reappraisal. Reappraisal has been generally considered an adaptive strategy, associated with healthiness and personal satisfaction (Aldao et al., 2010), that successfully modulates the affective state (Webb et al., 2012). Our analyses confirmed a positive relationship between EI and reappraisal (Hertel et al., 2009; Fernández-Berrocal and Extremera, 2016). Also, reappraisal has been long considered a protective factor over anxiety disorders (Hofmann et al., 2009). Our results confirm the negative relationship between anxiety and reappraisal. Importantly, the analyses also confirmed the role of anxiety and EI in suppression, although the direction, as expected, was the opposite compared with reappraisal. For what concerns EI (emotionality subscale), we have found that it is negatively correlated with the use of suppression. This further confirms previous observations according to which suppression comes with several costs in terms of physiological, cognitive, and emotional functioning (Nezlek and Kuppens, 2008; Brans, et al., 2013). Indeed, suppression has been associated with decreased emotional well-being and difficulties in the recognition and expression of emotions (Petrides, 2009). Thus, our study confirms previous investigations and suggests a relationship between low EI and maladaptive emotion regulation processes (Peña-Sarrionandia et al., 2015).

### Reappraisal and suppression are complementary strategies

Finally, our results suggest that reappraisal and suppression, respectively considered as adaptive and maladaptive strategies, are not mutually exclusive. In our model, the usage of one predicts the other, and they covary in a positive way. This means that individuals using one strategy also may be prone to use the other (Ferschmann et al., 2021), suggesting that an adaptive regulation may be built upon a wide range of strategies to be selected according to the context (Sahdra et al., 2020).

It is worth mentioning that previous studies separately assessed the association between one strategy and factors, such as anxiety, EI, and the use of other strategies. In our study, instead, we included all these variables in one unique model, thus enabling the evaluation of their relative influence. This model thus provides information concerning the joint role of these factors in predicting reappraisal or suppression. From our results, it is clear that EI and the use of the other strategy both outperform anxiety in their ability to predict the use of specific strategies (see the equations in the *Results* section).

## Conclusions and limitations

The purpose of this study was to decode individual differences in the use of reappraisal and suppression from structural brain networks, EI, and anxiety scores by using supervised and unsupervised machine-learning techniques. The result revealed a temporo-parahippocampal-orbitofrontal network predicting the habitual use of reappraisal. Differently, an insular network and a fronto-parietal-cerebellar network significantly predicted the use of suppression. In addition, the results reveal that EI and anxiety are both significant predictors of the use of reappraisal and suppression, although in opposite directions. Finally, the results suggest that reappraisal and suppression are complementary strategies, and the use each strategy is positively associated with the use of the other one. From the results of this study, we can conclude that independent neural circuits, EI, and anxiety jointly predict individual differences in the use of two important strategies of emotion regulation.

There are some limitations to note. Self-report questionnaires were used, and biases in these types of assessments due to the lack of awareness might have affected the results. Also, whereas the sample size of our study is in line with the recent literature focusing on similar issues in terms of structural neuroimaging data (Picó-Pérez et al., 2019; Baltruschat et al., 2021), it is smaller compared to investigations focusing on behavioural and psychological measures and predictors (Martin and Dahlen, 2005; Andrei et al., 2016; Nozaki, 2018). This warrants some caution when interpreting the individual differences at the level of psychological dimensions.

Furthermore, we only used structural data limited to grey matter. Future studies may want to extend these results to with matter and functional data. Finally, we used boosted regression trees to predict strategies usage. Other machine learning approaches are available and could have been used to predict the variable of interest. Because we do not know yet which algorithm works better for what kind of data, the present study may pave the way for additional methodological research on the comparison between different algorithms.
